# On the Strumous Diseases of Childhood

**Published:** 1890-10

**Authors:** Thomas More Madden

**Affiliations:** M. D., F. R. C. S. Ed.; Obstetric Physician; ex-President of the Royal Academy of Medicine, Ireland, and of the British Medical Association; formerly Vice-President British Gynæcological Society, etc.


### ON THE STRUMOUS DISEASES OF CHILDHOOD AND THEIR RELATION TO TUBERCLE.

By Thomas More Madden, M. D., F. R. C. S. Ed.

Obstetric Physician ; ex-President of the Royal Academy of Medicine, Ireland, and of the British Medical Association ; formerly Vice-President British Gynæcological Society, etc.

From the author's advance sheets of this valuable work, we extract the following :

During a long experience as physician to the first hospital for diseases of children, established in Ireland, with which I have been connected since its foundation in 1872, the increasing prevalence of the strumous and tubercular diseases of childhood, has been constantly brought under my clinical observation. The intimate connection and relation between these conditions was pointed out nearly a quarter of a century ago in my work on "Change of Climate," and was discussed in a paper of mine in the Transactions of the International Medical Congress of 1871, as well as last year in my article on "Puberty," in Dr. Keating's recently published American "Cyclopædia of Diseases of Children." I refer to these dates merely as evidence that the views embodied in the following brief recapitulation were not hastily formed nor without some experience of the subject referred to. The increasing proportion of strumous and tubercular affections which has been observed of late years in my wards in the Children's Hospital, is probably largely ascribable to the faulty diethetic and hygienic management of early childhood, and to the general substitution of artificial, and in many instances very unsuitable, preserved or tinned preparations, for that natural or fresh milk which in my opinion is essential for the healthy nutrition of children. As I formerly pointed out, and the observation is now more applicable than was the case ten years ago, the acute forms of tuberculosis, common during childhood, resemble the infective disease in their origin from a specific germ, whether generated in the body or introduced from without. The latter is probably the case in the tubercular diseases prevalent amongst the children of the poor, in whose dietary various forms of preserved milk foods now enter largely, as it seems difficult to conceive any certain guarantee that the cows furnishing the supply may not, in some cases, suffer from *perlsucht*, this disease being very prevalent and not materially affecting the quantity of milk. More recently, Professor Bollinger has shown that milk may prove infectious, whether taken from cows suffering from general or local tuberculosis; in his experiments only a few drops of undiluted milk from a tuberculous cow, proved sufficient to produce miliary tuberculosis in animals. Be the pathogenesis of tuberculosis what it may, however, there can, I think, be no question as to the fact that it is most frequently developed in patients who bear in their general constitutional condition, and more especially in their glandular system, the obvious imprint of the strumous diathesis. Nor is it to be wondered at, that in children thus constitutionally enfeebled, the struggle for existence between the invading specific micro-organisms and the blood corpuscles or leucocytes should almost invariably so speedily terminate in the fatal victory of the prolific bacilli of tubercle.

